# Supplementary material for: COVID-19 Pandemic: Escape of Pathogenic Variants and MHC Evolution
Source: Int J Mol Sci. 2022 Feb 28;23(5):2665. doi: 10.3390/ijms23052665 (PMC8910380; doi:10.3390/ijms23052665)
Supplement: Supplementary file 1 [file ijms-23-02665-s001.zip › ijms-1571499-supplementary.pdf]

COVID19 pandemic: Escape of pathogenic variants and MHC evolution

Pierre Pontarotti[1,2,3*], Julien Paganini[3*]

1) Evolutionary Biology team. MEPHI Aix Marseille Univ IRD, APHM.19-21 Boulevard Jean Moulin 13005 Marseille, France

2) SNC 5039 CNRS, France

3) Xegen 15 Rue Dominique Piazza, 13420 Gemenos, France.

*Corresponding authors: pierre.pontarotti@univ-amu.fr (Pierre Pontarotti) and Julien.paganini@xegen.fr (Julien Paganini)

List of Supplementary information

Supplementary File 1 : Model for the pathogen variant escape T cell memory as a motor of MHC evolution

Supplementary File S1: Model for the pathogen variant escape T cell memory as a motor of MHC evolution

This is a theoretical example for illustration or hypothesis purposes.

In a given population there are three alleles named: Allele 1, Allele 2, and Allele 3.

Each presents at a frequency of 33 percent.

Allele 1 is able to recognise three peptides (from a pathogen): A, B and B'.

Allele 2 is able to recognise three other peptides: C, D and D'.

Allele 3 is able to recognise three other peptides: E, F and rF '.

The immune memory for the part of the population carrying Allele 1 will be regarding peptide A (the TCR will recognise Allele 1 plus peptide A).

Another part of the population carrying Allele 1 will recognise the B peptide, and another part will recognise the B' peptide.

For Allele 2, part of the population will recognise peptide C, another will recognise peptide D, and another peptide D'.

For Allele 3, part of the population will recognise peptide E, another part will recognise peptide F, and another F'.

The virus evolves via a mutation in the region of the A peptide, so there will be no T protection for individuals with Allele 1 having anti A memory ,while there will be protection for individuals with Allele 1 having a memory against peptide B and B'.

All individuals with Allele 2 will be protected.

All individuals with Allele 3 will be protected.

Therefore people with Allele 1 will be disadvantaged compared to those with Alleles 2 and 3, and the frequency of Allele 1 or Alleles 2 and 3 will drop.

It can be theoretically stated that there will be a frequency of 16/42/42.

Immune evasion with a rare allele can even be included, so if there is a rare allele it will increase in frequency.

A new mutation of the virus occurs corresponding to the C peptide, so here it is the frequency of Allele 2 that will drop, while Allele 3 will increase in frequency.

There could, therefore, be a frequency of 20/20/60.

Let’s say that it stops at generation 1.

Starting again in generation 2, there is now a frequency of 20/20/60.

The epidemic starts again with a variant virus that will have a new antigenic peptide for Allele 1.

The following T cell memory repertoire will take place:

Allele 1 group 1 recognises A, group 2 recognises G, and group 3 recognises B’.

Allele 2 group 1 recognises D and group 2 recognises D’.

Allele 3 group 1 recognises E, group 2 recognises F, and group recognises 3 F’.

The virus (pathogen) evolves via mutation of a region which corresponds to the position of peptide F, so it is Allele 3 that will be disadvantaged and its frequency will drop. Hence, in this generation the frequencies will still vary.

In generations 2, 3, etc. epidemics may arise due to different viruses/ pathogen with the same outcome on the variation of the MHC allele frequency.
